# Associations of clinical context-specific ambiguity tolerance with burnout and work engagement among Japanese physicians: a nationwide cross-sectional study

**DOI:** 10.1186/s12909-024-05644-3

**Published:** 2024-06-14

**Authors:** Hirohisa Fujikawa, Takuya Aoki, Takayuki Ando, Junji Haruta

**Affiliations:** 1 Center for General Medicine Education, School of Medicine, Keio University, 35 Shinanomachi, Shinjuku-ku, Tokyo, 160-8582 Japan; 2 Department of Medical Education Studies, International Research Center for Medical Education, Graduate School of Medicine, The University of Tokyo, Bunkyo-ku, Tokyo, Japan; 3 Division of Clinical Epidemiology, The Jikei University School of Medicine, Minato-ku, Tokyo, Japan; 4 Section of Clinical Epidemiology, Department of Community Medicine, Graduate School of Medicine, Kyoto University, Sakyo-ku, Kyoto, Japan; 5 Medical Education Center, School of Medicine, Keio University, Shinjuku-ku, Tokyo, Japan

**Keywords:** Ambiguity tolerance, Tolerance for ambiguity, Tolerance of ambiguity, Clinical context, Burnout, Work engagement

## Abstract

**Purpose:**

Ambiguity tolerance specific to the clinical context – in contrast to ambiguity tolerance as a personality trait – may vary with experience and has received considerable attention. Although this tolerance appears to be related to burnout and work engagement, few studies have examined this association among physicians. Thus, we aimed to examine the relationships between clinical context-specific ambiguity tolerance, burnout, and work engagement among physicians in Japan.

**Methods:**

We conducted a nationwide cross-sectional study in Japan. We invited family physicians from 14 family medicine residency programs and physicians with specialties other than family medicine from monitors of an Internet survey company to participate in the study. We measured ambiguity tolerance in the clinical context using the Japanese version of the Tolerance of Ambiguity in Medical Students and Doctors (J-TAMSAD) scale, burnout using the Japanese version of the Burnout Assessment Scale (BAT-J), and work engagement using the Utrecht Work Engagement Scale (UWES). We performed a multivariable linear regression analysis to determine whether the J-TAMSAD scale score was associated with the BAT-J and UWES scores.

**Results:**

383 respondents were included in the analysis. After adjustment for possible confounders, clinical context-specific ambiguity tolerance showed a dose-dependent negative association with burnout (adjusted mean difference  -0.39, 95% confidence interval (CI) -0.56 to -0.22 for the highest J-TAMSAD score quartile compared with the lowest). Ambiguity tolerance in the clinical context also showed a dose-dependent positive association with work engagement (adjusted mean difference 0.83, 95% CI 0.49 to 1.16 for the highest J-TAMSAD score quartile compared with the lowest).

**Conclusions:**

Our study showed that tolerance for ambiguity in the clinical context was negatively associated with burnout, and positively associated with work engagement. These findings will be useful in developing interventions aimed at preventing burnout and promoting work engagement among physicians.

**Supplementary Information:**

The online version contains supplementary material available at 10.1186/s12909-024-05644-3.

## Background

In recent decades, widespread burnout among physicians has emerged as a critical issue worldwide [1]. In the U.S., the rate of burnout among physicians, about 50%, is higher than that of the general population, at about 30% [2, 3]. One in four medical trainees in the U.K. is at high risk of burnout [4]. The prevalence of burnout among Chinese physicians ranges from 67 to 88% [5]. A considerable body of literature has shown that physician burnout can have undesirable consequences: notably, it is associated with higher rates of turnover intention, an increase in medical errors, a decrease in patient experience, and a poorer quality of patient care [6–8]. These findings indicate a clear need to explore ways to prevent physician burnout as a means of protecting both the physicians themselves and their patients.

Work engagement – defined as a positive, emotionally motivated, and fulfilling state of work-related well-being – has attracted substantial attention in the field of occupational health psychology [9]. A worldwide search for ways to prevent burnout in recent years has led to the concept of work engagement, which is considered the antithesis of burnout [10]. Physician work engagement has positive associations with better job performance, fewer medical errors, and improved patient safety-related attitudes and behaviors [11–14]. Promoting work engagement among physicians therefore appears important.

Ambiguity is common in the practice of medicine, and the importance of nurturing tolerance for ambiguity has been promoted in recent years. Ambiguity is defined as a “lack of reliability, credibility, or adequacy” and tolerance for ambiguity as “the tendency to perceive ambiguous situations as desirable” [15, 16]. Although ambiguity and uncertainty are commonly used interchangeably, there is a distinct difference between them; ambiguity is a property of a stimulus, and ambiguity tolerance is present-oriented, whereas uncertainty is a response to a stimulus (i.e., ambiguity, complexity, and probability), and tolerance for uncertainty is future-oriented [16–18]. Tolerating ambiguity and knowing how to deal with it are prerequisites for the medical professional [19]. Studies have shown the importance of tolerance for ambiguity. For example, medical trainees in the U.S. who were tolerant of ambiguity were significantly less likely than those who were less tolerant of ambiguity to have negative attitudes toward the poor and underserved [20]. A recent study in Japan indicated that tolerance for ambiguity may be related to empathy among medical trainees [21]. Thus, tolerance of ambiguity is associated with range of desirable outcomes.

While several studies have examined the relationship between uncertainty tolerance and psychological well-being [22], only one U.S. study by Mangione et al. has explored whether tolerance for ambiguity is negatively associated with burnout [23]. However, the U.S. study was limited in three regards. First, the relationship between ambiguity tolerance and burnout was tested using correlations only. Second, the authors did not measure ambiguity tolerance in the clinical context. Because ambiguity tolerance in the clinical context may change with education, experience, or environment, as opposed to ambiguity tolerance as a personality trait and thus unlikely to change, research should focus on ambiguity tolerance specific to the clinical context [24]. Third, the study included medical students only. Therefore, examining the association between ambiguity tolerance specific to the clinical context and burnout in the postgraduate setting is a high priority. In addition, we are unaware of any study which aimed to verify the association between tolerance for ambiguity and work engagement, but this also warrants clarification.

Here, we investigated the association between clinical context-specific ambiguity tolerance and burnout among physicians. We included work engagement as a secondary outcome. Our aim was to provide insights into the role of ambiguity tolerance in occupational health and to contribute to improvements in postgraduate medical education.

## Methods

### Design, setting, and participants

The present study was conducted as part of a series of studies which explored tolerance of ambiguity and its related factors among Japanese physicians, conducted using a cross-sectional online survey across Japan. Because the series included research which compared the ambiguity tolerance of family physicians and non-family physicians (unpublished), we recruited physicians in two ways. First, physicians specializing in areas other than family medicine were recruited through an online survey company from among monitors registered with the company. Monitors with a specialty other than family medicine were randomly selected by the company and invited to participate. Second, since all the authors are family physicians, we asked directors of 14 family medicine residency programs, which varied in size and location, to distribute our online questionnaire in their programs. In the present study, family physicians were defined as “Japan Primary Care Association (JPCA; recognized certifying body for primary care physicians) certified primary care physicians, JPCA certified family physicians, general medicine specialists (accredited by the Japanese Medical Specialty Board), or trainees in these specialties,” and only family physicians who met this condition were included as participants with the specialty of family medicine. These family physicians work in a variety of settings, including inpatient, outpatient, and/or home-visit care. We adopted this strategy to recruit family physicians for two reasons. First, family physicians account for only a small percentage (less than 1%) of physicians in Japan [25], and the number of family physicians registered with the Internet survey company was small. Second, we anticipated that this strategy would allow us to collect as many family physicians as possible with the highest possible response rate.

Before participating in the study, all participants were informed that participation was voluntary and anonymous, and that not participating would not lead to any disadvantage. The response period was February 1 to 23, 2024. Participants received several reminder emails during the period.

### Measures

#### Primary outcome: burnout

The primary outcome was burnout, measured using the Japanese version of the Burnout Assessment Scale (BAT-J) [26–28]. The reliability and validity of this scale have been closely examined [26]. It includes 4 subscales with 23 items (exhaustion, 8 items (e.g., “At work, I feel mentally exhausted”), Cronbach’s alpha 0.93; mental distancing, 5 items (e.g., “I struggle to find any enthusiasm for my work”), Cronbach’s alpha 0.86; cognitive impairment, 5 items (e.g., “At work, I have trouble staying focused”), Cronbach’s alpha 0.93; emotional impairment, 5 items (e.g., “At work, I feel unable to control my emotions”), Cronbach’s alpha 0.91), with responses obtained using a five-point Likert scale ranging from never (1) to always (5). The scale is scored by averaging the scores of all 23 items and ranges between 1 and 5, with higher scores indicating a higher risk of burnout [26].

#### Secondary outcome: work engagement

The secondary outcome was work engagement, measured using the ultra-short version of the Utrecht Work Engagement Scale (UWES) [29, 30]. This scale has been well validated [29, 30]. It comprises three items (e.g., “At my work, I feel bursting with energy”), with responses obtained using a seven-point Likert scale ranging from 0 = never to 6 = always. The total score is obtained by averaging the scores for each item and ranges from 0 to 6, with higher scores indicating better work engagement [29, 30].

#### Explanatory variable: tolerance for ambiguity in the clinical context

We used the Japanese version of the Tolerance of Ambiguity in Medical Students and Doctors (J-TAMSAD) scale to assess tolerance for ambiguity specific to the clinical context [31, 32]. Previous research has shown the good reliability and validity of this scale [31]. It is an 18-item questionnaire, with responses obtained using a 5-point Likert scale (1 = strongly disagree to 5 = strongly agree). The total score (range: 0–100) is calculated using the following formula: J-TAMSAD total score = 25*(the average score of the 18 items − 1), with higher scores indicate greater tolerance for ambiguity [31]. The detail of this scale was presented in Supplementary file 1.

Because the J-TAMSAD scale was validated among medical students and residents of postgraduate year (PGY) 1/2 in a previous study, we considered whether the scale could be used in the current study population before the main multivariable analysis. First, the face validity was confirmed by all authors. Second, the Cronbach’s alpha value of the scale was 0.76, exceeding the acceptable criteria of internal consistency reliability (> 0.70) [33]. Thus, we decided to use the scale among our samples.

#### Covariates

Our regression models were adjusted for gender (female, male (reference category), or non-binary), PGY (3–6 (reference category), 7–15, 16–25, or ≥ 26), and specialty (family medicine (reference category), internal medicine and pediatrics, surgery medicine, or other departments) with reference to previous research. Scores of burnout and work engagement can be affected by gender, PGY, and specialty [34, 35]. Tolerance for ambiguity can be associated with PGY and specialty [32, 36]. For PGY, this classification was adopted with reference to its quartiles and the fact that PGY 3–6 correspond to senior residents.

### Statistical analysis

We used a multivariable linear regression model to examine whether the J-TAMSAD scale score was associated with the BAT-J or UWES scores. Since the assumption of linearity may not be met in the association between explanatory and outcome variables in our regression model, we categorized the J-TAMSAD scale score into quartiles [37]. We decided to perform complete case analysis because there were few participants with missing data. For all statistical analyses, we used a two-sided significance level of *P* < 0.05.

Previous literature on the sample size formula suggested a sample size per independent variable of ≥ 20 was needed for linear regression analyses [38]. Given that our model included 11 independent variables, we estimated a minimum sample size of 220. Statistical analyses were performed using SPSS Statistics version 29.0 (IBM Corp).

### Ethical considerations

In this study, participants were asked to read a description of the study at the beginning of the questionnaire and check the consent box to participate. The study was approved by the ethical committee of Keio University School of Medicine (approval number: 20231161).

## Results

**Fig. 1:**
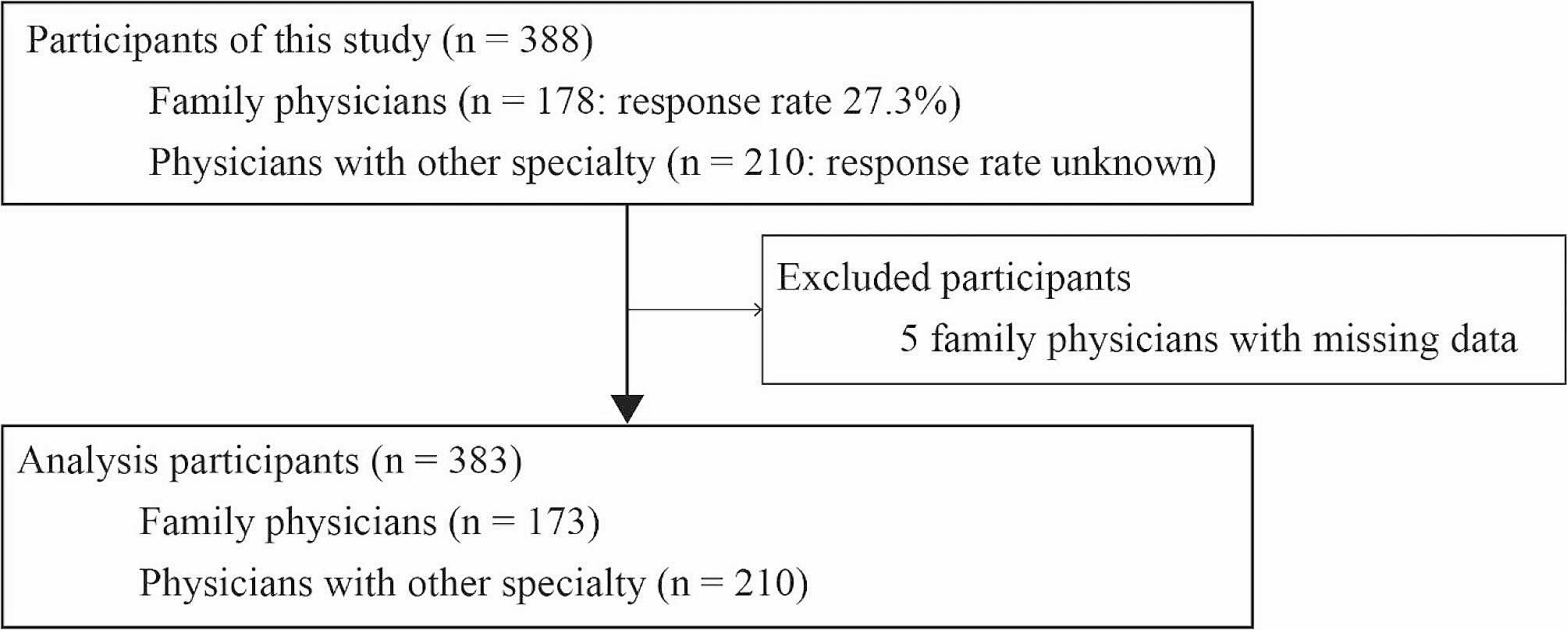
Participant flowchart

The participant flowchart is shown in Fig. 1. 178 (27.3%) of the 651 eligible family physicians completed the survey. 210 physicians in specialties other than family medicine also answered the questionnaire, although the response rate is unknown owing to the survey design (i.e., web survey) [39]. Of the 388 participants, we excluded 5 participants due to missing data, and analyzed the data of the remaining 383 participants. Table 1 shows the characteristics of the 383 participants. The majority of participants were male (78.6%), had a PGY of 7–15 (31.1%), and specialists in family medicine (46.4%).

**Table 1:** Characteristics of the study participants (*N* = 383)

Characteristic	Value
Gender, n (%)	
Female Male Others	81 (21.1) 301 (78.6) 1 (0.3)
PGYs, n (%)	
3–6 7–15 16–25 ≥ 26	68 (17.8) 119 (31.1) 100 (26.1) 96 (25.1)
Specialty, n (%)	
Family medicine Internal medicine Orthopedic surgery Surgery Psychiatry Pediatrics Urology Neurosurgery Dermatology Otorhinolaryngology Anesthesiology Ophthalmology Radiology Emergency medicine Obstetrics and gynecology Plastic surgery Rehabilitation Others	173 (46.4) 32 (8.6) 28 (7.5) 24 (6.4) 20 (5.4) 19 (5.1) 14 (3.8) 12 (3.2) 11 (2.9) 10 (2.7) 9 (2.4) 9 (2.4) 6 (1.6) 5 (1.3) 4 (1.0) 4 (1.0) 2 (0.5) 1 (0.3)
J-TAMSAD scale, mean (SD)	52.73 (9.71)
BAT-J, mean (SD)	2.52 (0.59)
UWES, mean (SD)	3.36 (1.11)

Abbreviations: BAT-J, Japanese version of the Burnout Assessment Scale; J-TAMSAD, Japanese version of the Tolerance of Ambiguity in Medical Students and Doctors; PGY, postgraduate year; SD, standard deviation; UWES, Utrecht Work Engagement Scale

Table 2 shows the results of a multivariable linear regression analysis of the associations between tolerance for ambiguity in the clinical context and burnout, and between tolerance for ambiguity in the clinical context and work engagement. After adjusting for possible confounders, ambiguity tolerance specific to the clinical context showed a dose-dependent negative association with the risk of burnout (i.e., increasing levels of ambiguity tolerance were associated with decreasing levels of burnout risk) (adjusted mean difference  -0.39, 95% confidence interval (CI) -0.56 to -0.22 for the highest J-TAMSAD score quartile compared with the lowest). Clinical context-specific ambiguity tolerance had a dose-dependent association with work engagement (i.e., increasing ambiguity tolerance was associated with increasing work engagement) (adjusted mean difference 0.83, 95% CI 0.49 to 1.16 for the highest J-TAMSAD score quartile compared with the lowest). We described the details of the analysis in Supplementary file 2.

**Table 2:** Associations between J-TAMSAD scale scores and BAT-J scores, and between J-TAMSAD scores and UWES scores (*N* = 383)

	Unadjusted mean difference (95% CI)	Adjusted^a^ mean difference (95% CI)
BAT-J^b^		
J-TAMSAD scale^d^		
Q1^e^ Q2^f^ Q3^g^ Q4^h^	Ref. -0.19 (-0.36 to -0.02)* -0.31 (-0.48 to -0.15)** -0.46 (-0.63 to -0.30)**	Ref. -0.16 (-0.33 to 0.01) -0.25 (-0.42 to -0.09)** -0.39 (-0.56 to -0.22)**
UWES^c^		
J-TAMSAD scale^d^		
Q1^e^ Q2^f^ Q3^g^ Q4^h^	Ref. 0.30 (-0.03 to 0.63) 0.37 (0.06 to 0.68)* 0.79 (0.47 to 1.10)**	Ref. 0.30 (-0.03 to 0.64) 0.38 (0.06 to 0.69)* 0.83 (0.49 to 1.16)**

Abbreviations: BAT-J, Japanese version of the Burnout Assessment Scale; CI, confidence interval; J-TAMSAD, Japanese version of the Tolerance of Ambiguity in Medical Students and Doctors; Q, quartile; UWES, Utrecht Work Engagement Scale

^a^ Adjusted for gender (female, male, or non-binary), postgraduate years (3–6, 7–15, 16–25, or ≥ 26), and specialty (family medicine, internal medicine and pediatrics, surgery medicine, or other departments)

^b^ Scores range from 1 to 5

^c^ Scores range from 0 to 6

^d^ Scores range from 0 to 100

^e^ 0–45.82

^f^ 45.83–51.37

^g^ 51.38–58.32

^h^ > 58.32.

* *p* < 0.05

** *p* < 0.01

## Discussion

This study showed that clinical context-specific ambiguity tolerance was negatively associated with burnout among Japanese physicians. Our results also demonstrated a significantly positive association between that tolerance of ambiguity in the clinical context and work engagement.

Our findings are consistent with previous research by Mangione et al. indicating that ambiguity tolerance had a negative correlation with burnout [23]. A possible mechanism proposed by Hancock et al. is that low ambiguity tolerance increases perceived psychological stress, leading to burnout [22]. Low tolerance for ambiguity can impede the formation of social relationships, performance in ambiguous situations, and the acquisition of complex ideas and skills [40, 41]. People with poor ambiguity tolerance seek to escape from ambiguous situations [40, 41], but in actual medical practice, there is a patient in front of them and thus escape is not possible. Thus, ambiguity tolerance may play a key role in regulating and controlling stress [42]: lower ambiguity tolerance is associated with greater psychological stress, which in turn likely leads to burnout. Nevertheless, the precise mechanism of this effect remains unknown, and further research into the causal relationship between tolerance for ambiguity and burnout is warranted.

The present study also revealed that ambiguity tolerance was positively associated with work engagement. Possible mechanisms for this association are as follows. First, physicians with greater ambiguity tolerance have greater cognitive flexibility [43], which leads to better coping with unexpected situations. This adaptability may allow them to work more effectively and increase their work engagement. Second, physicians with high tolerance for ambiguity may be less stressed when faced with ambiguous situations [44, 45], which may in turn allow them to be more motivated and maintain high levels of work engagement. However, since these are just hypotheses, more research needs to be done to determine how ambiguity tolerance increases work engagement.

The critical value of our findings is highlighted by the ongoing prevalence of physician burnout worldwide, with associated adverse effects on physicians themselves as well as on their patients. Interventions with the aim of increase ambiguity tolerance may reduce burnout and enhance work engagement among physicians. For example, previous studies suggest that reflective learning is likely to be key to fostering ambiguity tolerance [46, 47]. Reflection allows people to “reframe” ambiguous situations by shifting the focus away from negative associations with ambiguity, thereby providing them with flexible options [47]. Reflective learning diaries or reflection with mentors may aid in the development of ambiguity tolerance [47, 48]. As another example, recent studies have indicated that art classes, including visual thinking strategies, may be effective for nurturing tolerance of ambiguity among medical trainees [49, 50]. Art is said to be inherently ambiguous, because the artist’s intended expression can never be known with certainty by the viewer/audience [49]. While interpretations of art are subjective and can vary from person to person, visual thinking strategies encourage participants to engage with ambiguity by asking open-ended questions about the work and facilitate group discussions in which multiple interpretations are welcome [50]. This process may make participants more receptive to and tolerant of ambiguity. Our future studies will aim to assess physician burnout and work engagement longitudinally, while intervening in ambiguity tolerance through visual thinking strategies and other art activities.

To our knowledge, this is the first study to examine negative associations between ambiguity tolerance in the clinical context and burnout, and a positive association between clinical context-specific ambiguity tolerance and work engagement. The findings were based on data from a nationwide survey across Japan, and the scales used (i.e., J-TAMSAD scale, BAT-J, and UWES) are all well validated and widely used to measure the attributes of healthcare professionals [26–32]. Thus, our findings have relatively high validity. On the other hand, this study was conducted in Asia; given that responses to questionnaires on ambiguity tolerance, burnout, and work engagement may be influenced by culture, comparison with similar studies conducted outside Asia would provide a deeper understanding of the relationship between these three concepts.

### Limitations

Potential limitations of this study should be noted. First, it was conducted under a cross-sectional design, and accordingly did not allow any determination of causality or direction of relationships between tolerance for ambiguity and burnout, or between ambiguity tolerance and work engagement. Further longitudinal studies are required to confirm causality. Second, although we determined covariates with reference to previous research, unknown confounding factors can exist and influence the results. Third, study designs based around collaboration with online survey companies can raise concerns on the risk of sample bias. Fourth, the response rate of family physicians was a concern. It is possible that physicians with less ambiguity tolerance, greater risk of burnout, or less work engagement were less likely to participate in the study. If so, this could lead to underestimation of the relationship between ambiguity tolerance and burnout, and between tolerance of ambiguity and work engagement. Fifth, although our results are statistically significant, the clinical and occupational health relevance of the score difference remains unclear. The highest quartile adjusted mean difference value of 0.4 in this study roughly corresponds to the difference between the European BAT cutoff values for risk of burnout (2.59) versus being in burnout (3.02) [51]. Conversely, because the clinical cutoffs of the BAT-J and UWES have not been determined, further studies to interpret the scores of these scales may clarify the practical significance of our present results.

## Conclusions

Our study revealed that tolerance for ambiguity in the clinical context was negatively associated with burnout, and positively associated with work engagement among physicians. Our findings will be helpful for the development of interventions aimed at preventing physician burnout.

### Electronic supplementary material

Below is the link to the electronic supplementary material.

Supplementary Material 1

Supplementary Material 2

## Data Availability

Upon reasonable request, the corresponding author can provide the data sets generated and analyzed in the study.

## Abbreviations

J-TAMSAD scale: The Japanese version of the Tolerance of Ambiguity in Medical Students and Doctors scale
BAT-J: The Japanese version of the Burnout Assessment Scale
UWES: The Utrecht Work Engagement Scale
CI: Confidence interval
PGY: Postgraduate year

## References

[1] Rotenstein LS, Torre M, Ramos MA, Rosales RC, Guille C, Sen S (2018). Prevalence of burnout among physicians: a systematic review. JAMA.

[2] Shanafelt TD, Boone S, Tan L, Dyrbye LN, Sotile W, Satele D (2012). Burnout and satisfaction with work-life balance among US physicians relative to the general US population. Arch Intern Med.

[3] Shanafelt TD, Hasan O, Dyrbye LN, Sinsky C, Satele D, Sloan J et al. Changes in burnout and satisfaction with work-life balance in physicians and the general US working population between 2011 and 2014. Mayo Clin Proc. 2015;90(12):1600–13. 10.1016/j.mayocp.2015.08.023 26653297

[4] General Medical Council. National Training Survey 2023 results 2023. https://www.gmc-uk.org/-/media/documents/national-training-survey-2023-initial-findings-report_pdf-101939815.pdf.

[5] Lo D, Wu F, Chan M, Chu R, Li D (2018). A systematic review of burnout among doctors in China: a cultural perspective. Asia Pac Fam Med.

[6] Hodkinson A, Zhou A, Johnson J, Geraghty K, Riley R, Zhou A (2022). Associations of physician burnout with career engagement and quality of patient care: systematic review and meta-analysis. BMJ.

[7] Li CJ, Shah YB, Harness ED, Goldberg ZN, Nash DB (2023). Physician burnout and medical errors: exploring the relationship, cost, and solutions. Am J Med Qual.

[8] McKee KE, Tull A, Carmen MGd, Edgman-Levitan S (2020). Correlation of provider burnout with patient experience. J Patient Exp.

[9] Bakker AB, Schaufeli WB, Leiter MP, Taris TW (2008). Work engagement: an emerging concept in occupational health psychology. Work Stress.

[10] González-Romá V, Schaufeli WB, Bakker AB, Lloret S (2006). Burnout and work engagement: independent factors or opposite poles?. J Vocat Behav.

[11] Daugherty Biddison EL, Paine L, Murakami P, Herzke C, Weaver SJ (2016). Associations between safety culture and employee engagement over time: a retrospective analysis. BMJ Qual Saf.

[12] Mache S, Danzer G, Klapp BF, Groneberg DA (2013). Surgeons’ work ability and performance in surgical care: relations between organisational predictors, work engagement and work ability. Langenbecks Arch Surg.

[13] Prins JT, van der Heijden FMMA, Hoekstra-Weebers JEHM, Bakker AB, van de Wiel HBM, Jacobs B (2010). Burnout, engagement and resident physicians’ self-reported errors. Psychol Health Med.

[14] Scheepers RA, Arah OA, Heineman MJ, Lombarts KMJMH (2015). In the eyes of residents good supervisors need to be more than engaged physicians: the relevance of teacher work engagement in residency training. Adv Health Sci Educ Theory Pract.

[15] Budner S (1962). Intolerance of ambiguity as a personality variable. J Pers.

[16] Hillen MA, Gutheil CM, Strout TD, Smets EMA, Han PKJ (2017). Tolerance of uncertainty: conceptual analysis, integrative model, and implications for healthcare. Soc Sci Med.

[17] Carleton RN (2014). The intolerance of uncertainty construct in the context of anxiety disorders: theoretical and practical perspectives. Expert Rev Neurother.

[18] Furnham A, Marks J (2013). Tolerance of ambiguity: a review of the recent literature. Psychology.

[19] Schön DA (1983). The reflective practitioner: how professionals think in action.

[20] Wayne S, Dellmore D, Serna L, Jerabek R, Timm C, Kalishman S (2011). The association between intolerance of ambiguity and decline in medical studentsʼ attitudes toward the underserved. Acad Med.

[21] Fujikawa H, Aoki T, Son D, Hayashi M, Eto M. Association between tolerance for ambiguity specific to the clinical context and empathy in medical trainees: a multicenter cross-sectional study in Japan. Med Teach. 2024:46(4):512–8. 10.1080/0142159X.2023.2259065 37734453

[22] Hancock J, Mattick K (2020). Tolerance of ambiguity and psychological well-being in medical training: a systematic review. Med Educ.

[23] Mangione S, Chakraborti C, Staltari G, Harrison R, Tunkel AR, Liou KT (2018). Medical students’ exposure to the humanities correlates with positive personal qualities and reduced burnout: a multi-institutional U.S. survey. J Gen Intern Med.

[24] Bovier PA, Perneger TV (2007). Stress from uncertainty from graduation to retirement—a population-based study of Swiss physicians. J Gen Intern Med.

[25] Kato D, Ryu H, Matsumoto T, Abe K, Kaneko M, Ko M (2019). Building primary care in Japan: literature review. J Gen Fam Med.

[26] Sakakibara K, Shimazu A, Toyama H, Schaufeli WB (2020). Validation of the Japanese Version of the Burnout Assessment Tool. Front Psychol.

[27] Schaufeli WB, De Witte H, Desart S. Manual Burnout Assessment Tool (BAT). Internal report. KU Leuven, Belgium2020. https://burnoutassessmenttool.be.

[28] Schaufeli WB, Desart S, De Witte H (2020). Burnout Assessment Tool (BAT)—development, validity, and reliability. Int J Environ Res Public Health.

[29] Schaufeli WB, Shimazu A, Hakanen J, Salanova M, De Witte H (2019). An ultra-short measure for work engagement: the UWES-3 validation across five countries. Eur J Psychol Assess.

[30] Shimazu A, Schaufeli WB, Kosugi S, Suzuki A, Nashiwa H, Kato A (2008). Work engagement in Japan: validation of the Japanese version of the Utrecht Work Engagement Scale. Appl Psychol.

[31] Fujikawa H, Son D, Hayashi M, Kondo K, Eto M (2023). Translation, adaptation, and validation of the Tolerance of Ambiguity in Medical students and doctors (TAMSAD) scale for use in Japan. BMC Med Educ.

[32] Hancock J, Roberts M, Monrouxe L, Mattick K (2015). Medical student and junior doctors’ tolerance of ambiguity: development of a new scale. Adv Health Sci Educ Theory Pract.

[33] DeVellis RF (2017). Scale development: theory and applications.

[34] Matsuo T, Takahashi O, Kitaoka K, Arioka H, Kobayashi D (2021). Resident burnout and work environment. Intern Med.

[35] Rao S, Ferris TG, Hidrue MK, Lehrhoff SR, Lenz S, Heffernan J (2020). Physician burnout, engagement and career satisfaction in a large academic medical practice. Clin Med Res.

[36] Geller G, Tambor ES, Chase GA, Holtzman NA (1993). Measuring physicians’ tolerance for ambiguity and its relationship to their reported practices regarding genetic testing. Med Care.

[37] Katz MH (2011). Multivariable analysis: a practical guide for clinicians and public health researchers.

[38] Hair JF, Black WC, Babin BJ, Anderson RE (2019). Multivariate data analysis.

[39] Manfreda KL, Berzelak N, Vehovar V, Lovric M (2011). Nonresponse in web surveys. International Encyclopedia of Statistical Science.

[40] Tynan M. Multidimensional Tolerance Of Ambiguity: Construct Validity, Academic Success, And Workplace Outcomes [Doctoral dissertation]. Ames, IA: Iowa State University; 2020.

[41] Xu J, Ba Y (2022). Coping with students’ stress and burnout: learners’ ambiguity of tolerance. Front Psychol.

[42] Xu H, Tracey TJG (2014). The role of ambiguity tolerance in career decision making. J Vocat Behav.

[43] Weissenstein A, Ligges S, Brouwer B, Marschall B, Friederichs H (2014). Measuring the ambiguity tolerance of medical students: a cross-sectional study from the first to sixth academic years. BMC Fam Pract.

[44] Caulfield M, Andolsek K, Grbic D, Roskovensky L (2014). Ambiguity tolerance of students matriculating to U.S. medical schools. Acad Med.

[45] Iannello P, Mottini A, Tirelli S, Riva S, Antonietti A (2017). Ambiguity and uncertainty tolerance, need for cognition, and their association with stress. A study among Italian practicing physicians. Med Educ Online.

[46] English AR (2016). John Dewey and the role of the teacher in a globalized world: imagination, empathy, and ‘third voice’. Educ Philos Theory.

[47] Stephens GC, Sarkar M, Lazarus MD (2022). Medical student experiences of uncertainty tolerance moderators: a longitudinal qualitative study. Front Med (Lausanne).

[48] Nevalainen MK, Mantyranta T, Pitkala KH (2010). Facing uncertainty as a medical student—a qualitative study of their reflective learning diaries and writings on specific themes during the first clinical year. Patient Educ Couns.

[49] Bentwich ME, Gilbey P (2017). More than visual literacy: art and the enhancement of tolerance for ambiguity and empathy. BMC Med Educ.

[50] Cerqueira AR, Alves AS, Monteiro-Soares M, Hailey D, Loureiro D, Baptista S (2023). Visual thinking strategies in medical education: a systematic review. BMC Med Educ.

[51] Schaufeli WB, De Witte H, Hakanen JJ, Kaltiainen J, Kok R (2023). How to assess severe burnout? Cutoff points for the Burnout Assessment Tool (BAT) based on three European samples. Scand J Work Environ Health.

