# Supplementary material for: Associations of clinical context-specific ambiguity tolerance with burnout and work engagement among Japanese physicians: a nationwide cross-sectional study
Source: BMC Med Educ. 2024 Jun 14;24:660. doi: 10.1186/s12909-024-05644-3 (PMC11179221; doi:10.1186/s12909-024-05644-3)
Supplement: Supplementary file 1 — Supplementary Material 1 [file 12909_2024_5644_MOESM1_ESM.docx]

The Japanese version of the Tolerance of Ambiguity in Medical Students and Doctors (J-TAMSAD) scale (BMC Med Educ. 2023;23(1):405.)

| Dimension | No. | Items (as in original English version) |
| --- | --- | --- |
| D5 | 1 | I have a lot of respect for consultants who always come up with a definite answer* |
| D1 | 2 | I would be comfortable if a clinical teacher set me a vague assignment or task |
| D1 | 3 | A good clinical teacher is one who challenges your way of looking at clinical problems |
| D5 | 4 | I think in medicine it is important to know exactly what you are talking about at all times* |
| D2 | 5 | I feel comfortable that in medicine there is often no right or wrong answer |
| D1 | 6 | A patient with multiple diseases would make a doctor’s job more interesting |
| D2 | 7 | The unpredictability of a patient’s response to medication would bring welcome complexity to a doctor’s role |
| D5 | 8 | Being confronted with contradictory evidence in clinical practice makes me feel uncomfortable* |
| D2 | 9 | I like the mystery that there are some things in medicine we’ll never know |
| D3 | 10 | Variation between individual patients is a frustrating aspect of medicine* |
| D3 | 11 | I find it frustrating when I can’t find the answer to a clinical question* |
| D4 | 12 | No matter how complicated the situation, a good doctor will be able to arrive at a yes or no answer* |
| D5 | 13 | I feel uncomfortable when textbooks or experts are factually incorrect* |
| D4 | 14 | There is really no such thing as a clinical problem that can’t be solved* |
| D1 | 15 | I like the challenge of being thrown in the deep end with different medical situations |
| D1 | 16 | It is more interesting to tackle a complicated clinical problem that to solve a simple one |
| D1 | 17 | I enjoy the process of working with a complex clinical problem and making it more manageable |
| D4 | 18 | To me, medicine is black and white* |

Note: D1 = “Likes complicated, challenging, and vague situations in medical practice” (Cronbach’s alpha 0.75); D2 = “Likes the mystery that is medicine” (Cronbach’s alpha 0.71); D3 = “Tolerance for medical settings without a single solution” (Cronbach’s alpha 0.54); D4 = “Tolerance for things that are not black or white in medicine” (Cronbach’s alpha 0.60); D5 = “Tolerance for controversial circumstances in medical practice” (Cronbach’s alpha 0.52); * Reverse items
