# Supplementary material for: Associations of clinical context-specific ambiguity tolerance with burnout and work engagement among Japanese physicians: a nationwide cross-sectional study
Source: BMC Med Educ. 2024 Jun 14;24:660. doi: 10.1186/s12909-024-05644-3 (PMC11179221; doi:10.1186/s12909-024-05644-3)
Supplement: Supplementary file 2 — Supplementary Material 2 [file 12909_2024_5644_MOESM2_ESM.docx]

Supplementary file 2: The results of the multivariable regression analysis to examine the associations of clinical context-specific ambiguity tolerance with burnout and work engagement

|  | Unadjusted mean difference (95% CI) | Adjusted mean difference (95% CI) |
| --- | --- | --- |
| BAT-J^a^ |  |  |
| J-TAMSAD scale Q1^c^ | Ref. | Ref. |
| J-TAMSAD scale Q2^d^ | -0.19 (-0.36 to -0.02)* | -0.16 (-0.33 to 0.01) |
| J-TAMSAD scale Q3^e^ | -0.31 (-0.48 to -0.15)** | -0.25 (-0.42 to -0.09)** |
| J-TAMSAD scale Q4^f^ | -0.46 (-0.63 to -0.30)** | -0.39 (-0.56 to -0.22)** |
| PGY 3–6 |  | Ref. |
| PGY 7–15 |  | -0.28 (-0.45 to -0.11)** |
| PGY 16–25 |  | -0.42 (-0.60 to -0.24)** |
| PGY ≥ 26 |  | -0.58 (-0.77 to -0.39)** |
| Male |  | Ref. |
| Female |  | -0.09 (-0.24 to 0.05) |
| Non-binary |  | 0.33 (-0.77 to 1.42) |
| Family medicine |  | Ref. |
| Internal medicine and pediatrics |  | 0.20 (0.01 to 0.39)* |
| Surgery medicine |  | 0.21 (0.05 to 0.37)** |
| Other departments |  | 0.12 (-0.07 to 0.30) |
| UWES^b^ |  |  |
| J-TAMSAD scale Q1^c^ | Ref. | Ref. |
| J-TAMSAD scale Q2^d^ | 0.30 (-0.03 to 0.63) | 0.30 (-0.03 to 0.64) |
| J-TAMSAD scale Q3^e^ | 0.37 (0.06 to 0.68)* | 0.38 (0.06 to 0.69)* |
| J-TAMSAD scale Q4^f^ | 0.79 (0.47 to 1.10)** | 0.83 (0.49 to 1.16)** |
| PGY 3–6 |  | Ref. |
| PGY 7–15 |  | 0.29 (-0.04 to 0.62) |
| PGY 16–25 |  | 0.38 (0.04 to 0.73)* |
| PGY ≥ 26 |  | 0.39 (0.02 to 0.76)* |
| Male |  | Ref. |
| Female |  | -0.07 (-0.36 to 0.21) |
| Non-binary |  | -0.44 (-2.59 to 1.70) |
| Family medicine |  | Ref. |
| Internal medicine and pediatrics |  | 0.23 (-0.14 to 0.60) |
| Surgery medicine |  | -0.07 (-0.38 to 0.23) |
| Other departments |  | 0.21 (-0.15 to 0.57) |

Abbreviations: BAT-J, Japanese version of the Burnout Assessment Scale; CI, confidence interval; J-TAMSAD, Japanese version of the Tolerance of Ambiguity in Medical Students and Doctors; Q, quartile; UWES, Utrecht Work Engagement Scale.

^a^ Scores range from 1 to 5

^b^ Scores range from 0 to 6

^c^ 0–45.82

^d^ 45.83–51.37

^e^ 51.38–58.32

^f^ 58.33–100

* p < 0.05

** p < 0.01
